# Is the ACS-NSQIP Risk Calculator Accurate in Predicting Adverse Postoperative Outcomes in the Emergency Setting? An Italian Single-center Preliminary Study

**DOI:** 10.1007/s00268-020-05705-w

**Published:** 2020-07-24

**Authors:** Giovanni Scotton, Giulio Del Zotto, Laura Bernardi, Annalisa Zucca, Susanna Terranova, Stefano Fracon, Lucia Paiano, Davide Cosola, Alan Biloslavo, Nicolò de Manzini

**Affiliations:** Department of General Surgery, ASUGI, Cattinara Hospital, Strada di Fiume 447, 34149 Trieste TS, Italy

## Abstract

**Background:**

The ACS-NSQIP surgical risk calculator (SRC) is an open-access online tool that estimates the chance for adverse postoperative outcomes. The risk is estimated based on 21 patient-related variables and customized for specific surgical procedures. The purpose of this monocentric retrospective study is to validate its predictive value in an Italian emergency setting.

**Methods:**

From January to December 2018, 317 patients underwent surgical procedures for acute cholecystitis (*n* = 103), appendicitis (*n* = 83), gastrointestinal perforation (*n* = 45), and intestinal obstruction (*n* = 86). Patients’ personal risk was obtained and divided by the average risk to calculate a personal risk ratio (RR). Areas under the ROC curves (AUC) and Brier score were measured to assess both the discrimination and calibration of the predictive model.

**Results:**

The AUC was 0.772 (95%CI 0.722–0.817, *p* < 0.0001; Brier 0.161) for serious complications, 0.887 (95%CI 0.847–0.919, *p* < 0.0001; Brier 0.072) for death, and 0.887 (95%CI 0.847–0.919, *p* < 0.0001; Brier 0.106) for discharge to nursing or rehab facility. Pneumonia, cardiac complications, and surgical site infection presented an AUC of 0.794 (95%CI 0.746–0.838, *p* < 0.001; Brier 0.103), 0.836 (95%CI 0.790–0.875, *p* < 0.0001; Brier 0.081), and 0.729 (95%CI 0.676–0.777, *p* < 0.0001; Brier 0.131), respectively. A RR > 1.24, RR > 1.52, and RR > 2.63 predicted the onset of serious complications (sensitivity = 60.47%, specificity = 64.07%; NPV = 81%), death (sensitivity = 82.76%, specificity = 62.85%; NPV = 97%), and discharge to nursing or rehab facility (sensitivity = 80.00%, specificity = 69.12%; NPV = 95%), respectively.

**Conclusions:**

The calculator appears to be accurate in predicting adverse postoperative outcomes in our emergency setting. A RR cutoff provides a much more practical method to forecast the onset of a specific type of complication in a single patient.

## Introduction

Acute non-traumatic abdominal pain is a common symptom in the emergency department, accounting for up to 5–6.6% of all visits [1, 2]. Similar results are consistent with data published by large Italian series, which report acute abdominal pain as the leading symptom in 5.76–9.1% of the total adult emergency department visits [3, 4]. Of hospital admissions for acute abdominal pain, biliary colic and cholecystitis account for as many as 18% of the cases, followed by appendicitis (15%) and diverticulitis (11%) [3]. Overall, these three diagnoses represent nearly half of all hospital admission.

Many of these patients require an emergency surgical treatment, and postoperative survival and complication rate may be severely influenced by the patients’ characteristics and preexisting comorbidities. Traditionally, patients are presented with a risk estimation based on a summary of published data and the surgeon’s personal experience, which insufficiently reflect the patient’s personal risk assessment. An accurate and individualized surgical risk stratification occupies a central position in the surgical decision making process and represents an essential element in the preoperative informed consent process, providing the patient with ample information about possible outcomes, alternatives in treatment, and relative risks [5].

The American College of Surgeons National Surgical Quality Improvement Program (ACS-NSQIP) collects clinical data on preoperative risk factors (patient demographics and comorbidities) and 30-day postoperative complications. Previously, a risk calculator was built using data collected from over 4.3 million operations from 780 hospitals in the USA participating in ACS-NSQIP from 2013 to 2017. It was designed to estimate patient specific risks of more than 2500 surgical procedures within the first month after surgery [6, 7]. The risk calculator uses 20 patient specific variables (e.g., age, ASA class, BMI) and the planned procedure (CPT code) to make logistic model-based predictions for 18 different procedure-specific outcomes within 30 days following surgery. The procedure-specific variable (current procedural terminology or CPT code) represents the strongest predictor in the risk calculation and is the mechanism by which SRC predictions can be effectively made for a wide range of operations [7]. The risk standardization based on the CPT code makes moreover these results comparable and allows their analysis within a single group, regardless of the variability in morbidity and mortality associated with the different procedures.

There is a paucity of data about the applicability of the ACS-NSQIP surgical risk calculator (SRC) in the emergency general surgery. A recent review examined all the existing risk stratification tools which can be applied to the emergency surgical care [8]. The authors considered the ACS-NSQIP SRC as reasonably accurate in terms of estimation of postoperative death and complication in the emergency setting, with readily obtainable objective data, which can be used in the early phases of decision making and care. Others confirmed that the ACS-NSQIP SRC accurately predicted complications after emergency procedures overall, but demonstrated a great variability in performance between procedure types [9].

In our view, the application of the SRC in the emergency setting should be validated also in populations outside the USA, where the calculator was created. The purpose of this monocentric study is to test the calculator’s predictive value in an Italian emergency setting. Furthermore, it aims to provide some practical suggestions to simplify its use for a single patient, such as the introduction of a risk ratio (RR) cutoff.

## Materials and methods

### Study design and data collection

The study was a single-institution retrospective review of patients treated in the General Surgery Department of Trieste’s University Hospital. Ethical committee approval was obtained according to the Italian law, and the study was conducted in accordance with Good Clinical Practice. Were considered for inclusion in the study all the patients aged 18 or older, admitted to our institution from January 2018 to the end of December 2018 with diagnosis of acute cholecystitis, appendicitis, non-traumatic gastrointestinal perforation or bowel obstruction, and in which the indication to emergency surgical procedure was considered as first choice of treatment or as salvage surgery when previous non-operative management failed (e.g., antibiotic treatment, percutaneous drainage, stenting). Non-traumatic gastrointestinal perforation included gastroduodenal perforation due to peptic ulcer disease, small bowel perforation, and colonic perforation with both neoplastic (colorectal cancer) and non-neoplastic (perforated diverticular disease) etiology. Bowel obstruction included small bowel obstruction caused by adhesions, tumors, gallstone ileus or foreign bodies, and large bowel obstruction caused by colorectal cancer. Exclusion criteria were: patients <18, bowel obstruction or perforation as a complication of abdominal wall hernias, bowel obstruction or perforation which occurred as a postoperative complication, multiple simultaneous surgical procedures (more than one CPT code), missing preoperative or postoperative data.

Data regarding demographic, surgical procedures, and complications were collected by retrospectively reviewing the prospectively maintained institutional databases. Missing data were completed by revision of the electronic medical records by three members of the research team. Data on postoperative complications were captured from medical documentation recorded within 30 days following surgery (discharge letters, laboratory analysis, follow-up surgical examinations, notes, specialty consultations, and imaging). A unique code was assigned to each patient meeting the inclusion criteria, and records were maintained in a designated database, encrypted, and password protected.

Individual patient characteristics were manually entered into the ACS-NSQIP SRC including procedure CPT code, age group, sex, functional status, emergency case, ASA class, steroid use for chronic condition, ascites within 30 days prior to surgery, systemic sepsis within 48 h prior to surgery, ventilator dependent, disseminated cancer, diabetes, hypertension requiring medication, congestive heart failure within 30 days prior to surgery, dyspnea, current smoker within 1 year, history of severe chronic obstructive pulmonary disease (COPD), dialysis, acute renal failure, and BMI (for definitions visit https://riskcalculator.facs.org/RiskCalculator/PatientInfo.jsp). For the question “Are there other potential appropriate treatment options?” “none” was systematically selected, and “Surgeon Adjustment of Risk” was always left on “1—No adjustment necessary.”

The estimated patient personal risks (“Your risk”) for each postoperative adverse event were recorded as a percentage, including serious complication (cardiac arrest, myocardial infarction, pneumonia, progressive renal insufficiency, acute renal failure, PE, DVT, return to the operating room, deep incisional SSI, organ space SSI, systemic sepsis, unplanned intubation, UTI, and wound disruption), any complication (superficial incisional SSI, deep incisional SSI, organ space SSI, wound disruption, pneumonia, unplanned intubation, PE, ventilator >48 h, progressive renal insufficiency, acute renal failure, UTI, stroke, cardiac arrest, myocardial infarction, DVT, return to the operating room, and systemic sepsis), pneumonia, cardiac complication (cardiac arrest or MI), surgical site infection (SSI), urinary tract infection (UTI), venous thromboembolism (VTE), renal failure (progressive renal insufficiency or acute renal failure), readmission, return to OR, death, discharge to nursing or rehab facility, and predicted length of hospital stay.

### Statistical analysis

Demographics and patients’ characteristics were summarized as mean ± standard deviation (SD) for continuous variables and percentage value for categorical variables. The length of postoperative hospital stay was expressed as median ± interquartile range (IQR). For a preliminary analysis, the estimated patient personal risk was divided by the “average risk” provided by the SRC to calculate a personal risk ratio (RR, RR > 1 was defined as above average risk and RR ≤ 1 as below average risk). The Chi-square test (or Fisher’s exact test, when appropriate) was then used to determine significant differences in the observed complications rate between the RR ≤ 1 and RR > 1 groups.

Predictive performance of the calculator was studied by assessing discrimination and calibration. Discrimination measures how well a regression model differentiates patients at higher risk of having an event (e.g., serious complication) from those at lower risk, using a set of predictor variables (e.g., age, BMI, smoker, comorbidities). It permits to characterize the risk of one patient when compared to the risk of another (e.g., patient A has three times the risk of patient B), without considering the absolute risk of the two patients*.* For an example, patient A could have an absolute risk of 20% and patient B of 60%, or patient A an absolute risk of 2% and patient B of 6%; in both cases the relative risk is the same, but the absolute risk changes enormously. As a result, discrimination alone is insufficient to assess the prediction capability of a model because it provides no information regarding whether the overall magnitude of risk is predicted accurately.

Calibration conversely measures the ability of one model to assign accurate absolute risk estimates, analyzing if the absolute number of observed events is statistically comparable to the absolute number of events predicted by the model [10].

In our study, discrimination was characterized using the area under (AUC) the receiver operating characteristic (ROC) curve or c-statistic. The AUC is scored between 1.0 (perfectly discriminating model) and 0.5 (no better than chance). Discrimination was considered poor with an AUC of 0.6–0.69, adequate with an AUC of 0.7–0.79, strong with an AUC of 0.8–0.89, and excellent with an AUC of 0.9–1.0. A sample size of 250 patients (alpha 0.05, power 90%) was calculated to show that an AUC of 0.7 differs significantly from the null hypothesis value 0.5.

The Brier’s score was utilized to assess calibration. This score is defined as the average squared difference between the predicted probabilities and the observed rate of binary outcome and has the benefit of being influenced by both discrimination and calibration. Brier’s score values range between 0 and 1, with 0 indicating a perfect model of prediction. A risk prediction model with a score of 0.25 or higher is considered non-informative [11]. Both c-statistic and Brier’s scores are statistical tools that were used in the original validation of the ACS-NSQIP SRC [6].

The previously calculated personal RRs were used to generate additional ROC curves for three specific outcomes of interest: serious complications, death, and discharge to nursing or rehab facility. By referring to these curves, a range of all possible RR cutoff points was chosen to keep both sensitivity and specificity values at least >60%. We arbitrarily assumed that a sensitivity and specificity simultaneous value of minimum >60% was indispensable to consider the RR cutoff range as informative. To calculate sensitivity and specificity, we then chose the intermediate value of this range. Positive and negative predicted values were then calculated.

For all the presented statistical analysis, *p* values <0.05 were considered statistically significant. Statistical analyses were performed using IBM SPSS Statistics version 21 (IBM Corporation, Armonk, NY) and MedCalc Statistical Software version 17.2 (MedCalc Software bvba, Ostend, Belgium).

## Results

From January 2018 to December 2018, a total of 360 patients were submitted to emergency surgical procedure due to acute cholecystitis, appendicitis, bowel obstruction, or gastrointestinal perforation. Thirty-four patients who underwent surgery for bowel obstruction or perforation as a complication of abdominal wall hernias were excluded from the study. Similarly, we excluded 9 patients operated for bowel obstruction or perforation which occurred as a postoperative complication, and 5 patient submitted to multiple procedures (e.g., simultaneous appendectomy and cholecystectomy). As a result, a total of 317 patients were included in the study. Data collection was complete in 100% of considered patients.

Preoperative diagnoses are reported in Table 1. Surgeries eligible for inclusion comprised open and laparoscopic procedures with specific CPT code, as summarized in Table 2. Patients’ demographics, preoperative characteristics, and observed complications are summarized in Table 3. UTI, VTE, and readmission were excluded from further analysis due to lack of observed events. The personal risk ratios (RR) were calculated and used to determine significant differences in observed complications rate between the RR ≤ 1 and RR > 1 groups (Table 4).

**Table 1:** Admission diagnosis

Diagnosis	*n*
Acute cholecystitis	103
Appendicitis	83
Bowel obstruction Small bowel obstruction Colorectal (neoplastic)	86 52 34
Gastrointestinal perforation Gastroduodenal Colorectal (diverticular disease) Colorectal (neoplastic) Small bowel	45 18 17 8 2
Total	317

**Table 2:** Surgical procedures

CPT code	Procedure	*n*
43632	Gastrectomy, partial, distal; with gastrojejunostomy	2
43840	Gastrorrhaphy, suture of perforated duodenal or gastric ulcer, wound, or injury	16
44005	Enterolysis (freeing of intestinal adhesion) (separate procedure)	39
44120	Enterectomy, resection of small intestine; single resection and anastomosis	12
44140	Colectomy, partial; with anastomosis	8
44143	Colectomy, partial; with end colostomy and closure of distal segment (Hartmann type procedure)	12
44146	Colectomy, partial; with coloproctostomy (low pelvic anastomosis), with colostomy	2
44150	Colectomy, total, abdominal, without proctectomy; with ileostomy or ileoproctostomy	3
44151	Colectomy, total, abdominal, without proctectomy; with continent ileostomy	2
44160	Colectomy, partial, with removal of terminal ileum with ileocolostomy	16
44180	Laparoscopy, surgical, enterolysis (freeing of intestinal adhesion) (separate procedure)	2
44204	Laparoscopy, surgical; colectomy, partial, with anastomosis	1
44208	Laparoscopy, surgical; colectomy, partial, with anastomosis, with coloproctostomy (low pelvic anastomosis) with colostomy	1
44320	Colostomy or skin level cecostomy	12
44604	Suture of large intestine (colorrhaphy) for perforated ulcer, diverticulum, wound, injury or rupture (single or multiple perforations); without colostomy	1
44950	Appendectomy	2
44960	Appendectomy; for ruptured appendix with abscess or generalized peritonitis	21
44970	Laparoscopy, surgical, appendectomy	59
45562	Exploration, repair, and presacral drainage for rectal injury;	1
47562	Laparoscopy, surgical; cholecystectomy	83
47600	Cholecystectomy	21
	Tot	317

*CPT code* current procedural terminology code

**Table 3:** Demographics and observed complications

Patient characteristics (*n* = 317)	Distribution	Observed complications	*n* (%)
Age, mean	61.23 (SD 20.32)	Serious complication	86 (27.1%)
Sex, males,* n* (%)	175 (55.2%)	Any complication	127 (40.1%)
ASA class, mean	1.82 (SD 0.82)	Pneumonia	41 (12.9%)
1, *n* (%)	136 (42.9%)	Cardiac complication	34 (10.7%)
2, *n* (%)	108 (34.0%)	SSI	53 (16.7%)
3, *n* (%)	69 (21.8%)	UTI	9 (2.8%)
4, *n* (%)	4 (1.3%)	VTE	2 (0.6%)
Height in meters, mean	1.69 (SD 0.09)	Renal failure	21 (7.2%)
Weight in kg, mean	72.88 (SD 14.92)	Readmission	8 (2.5%)
Functional Status		Return to OR	23 (7.2%)
Independent, *n* (%)	239 (75.4%)	Death	29 (9.2%)
Partially dependent, *n* (%)	72 (22.7%)	Discharge to nursing or rehab facility	45 (14.2%)
Totally dependent, *n* (%)	6 (1.9%)	Length of hospital stay, mean (days)	14.1 (SD 19.7)
Emergency case, *n* (%)	317 (100%)		
Steroid Use, *n* (%)	8 (2.5%)		
Ascites, *n* (%)	0 (0%)		
Sepsis within 48 h, *n* (%)	103 (32.5%)		
Ventilated, *n* (%)	0 (0%)		
Diabetes, *n* (%)	92 (29.0%)		
Hypertension, *n* (%)	97 (30.6%)		
CHF, *n* (%)	11 (3.5%)		
Dyspnea, *n* (%)	19 (6%)		
Smoker, *n* (%)	52 (16.4%)		
COPD, *n* (%)	17 (5.4%)		
Dialysis, *n* (%)	5 (1.6%)		
Acute renal failure, *n* (%)	7 (2.2%)		

*CHF* chronic heart failure,* COPD* chronic obstructive pulmonary disease,* SSI* surgical site infection,* UTI* urinary tract infection,* VTE* venous thromboembolism

**Table 4:** Observed complications rate in RR ≤ 1 and RR > 1

Observed complications	Complication rate in RR ≤ 1	Complication rate in RR > 1	*p* (Fisher exact test)
Serious complication	15.6% (21/135)	35.7% (65/182)	<0.001
Any complication	27.6% (37/134)	49.2% (90/183)	<0.001
Pneumonia	5.2% (8/153)	20.1% (33/164)	<0.001
Cardiac complication	4.6% (8/175)	18.3% (26/142)	<0.001
SSI	15.9% (32/201)	18.1% (21/116)	0.641
Renal failure	1.9% (4/209)	16.7% (18/108)	<0.001
Return to OR	5.2% (8/153)	9.1% (15/164)	0.200
Death	2.3% (4/176)	17.7% (25/141)	<0.001
Discharge to nursing or rehab facility	1.5% (2/137)	23.9% (43/180)	<0.001

*RR* risk ratio,* SSI* surgical site infection

**Fig. 1:**
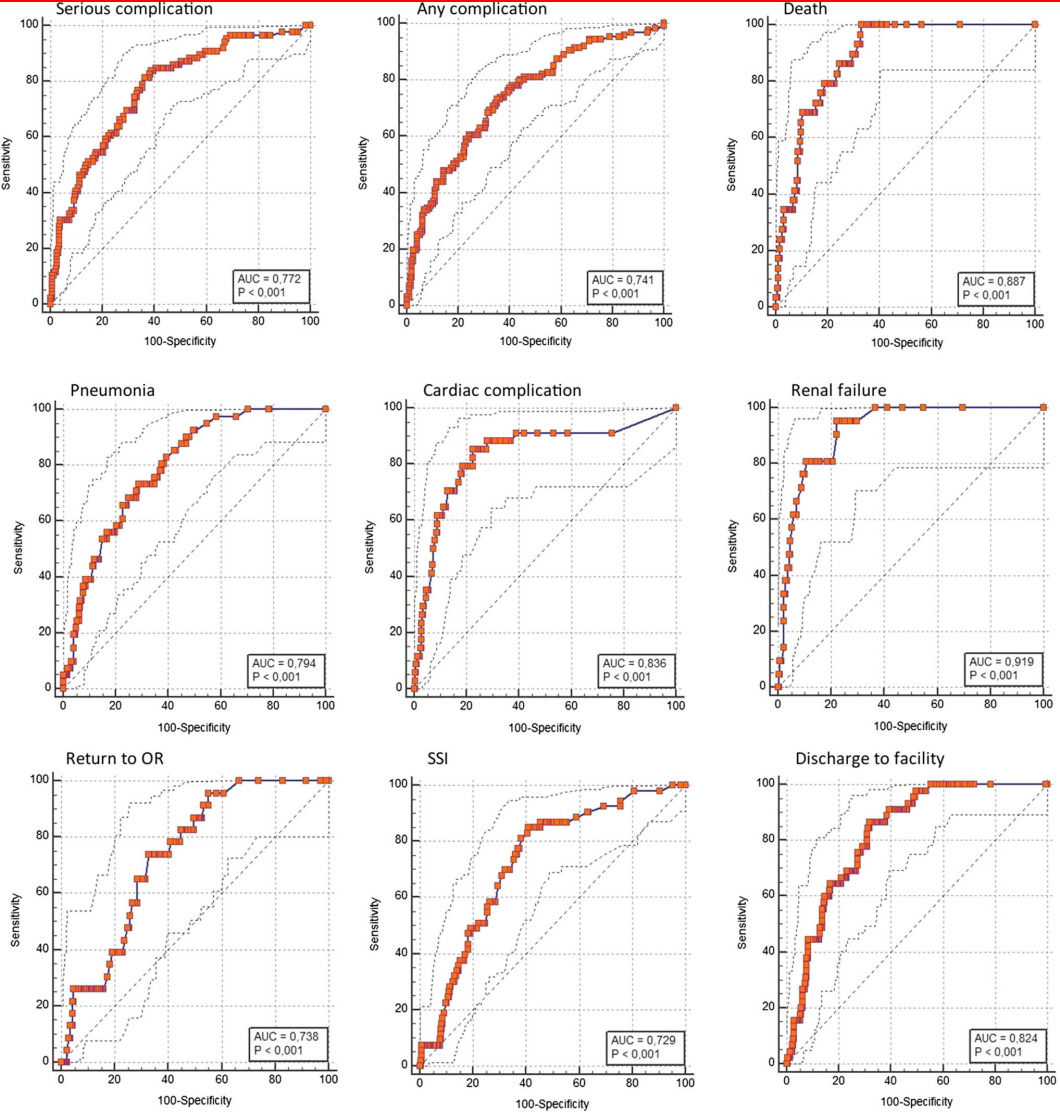
ROC curves

Discriminative performance was excellent for renal failure with an AUC of 0.919 (95% confidence interval 0.881–0.947, *p* < 0.0001). Death (AUC = 0.887, 95%CI 0.847–0.919, *p* < 0.0001), discharge to nursing or rehab facility (AUC = 0.824, 95%CI 0.777–0.864, *p* < 0.001), and cardiac complications (AUC = 0.836, 95%CI 0.790–0.875, *p* < 0.0001) showed strong discriminative performance. Any complication (AUC = 0.741, 95%CI 0.690–0.789, *p* < 0.0001), pneumonia (AUC = 0.794, 95%CI 0.746–0.838, *p* < 0.001), SSI (AUC = 0.729, 95%CI 0.676–0.777, *p* < 0.0001), return to the OR (AUC = 0.738, 95%CI 0.686–0.785, *p* < 0.0001) had adequate discriminative performance (Fig. 1). The median observed length of stay was 7 (IQR 4–12) days, compared to a predicted one of 6.5 (IQR 1.5–13) days (Mood’s median test, *p* = 0.695).

Brier score was 0.066 for return to OR, 0.072 for death, 0.081 for cardiac complication, 0.103 for pneumonia, 0.106 for discharge to nursing or rehab facility, 0.131 for SSI, 0.161 for serious complication, and 0.200 for any complication. Brier score was not informative for renal failure, with a value of 0.689 (Table 5).

**Table 5:** Discrimination and calibration assessment

Complications	AUC	Brier score
Renal failure	0.919 (*p* < 0.0001)	0.689
Death	0.887 (*p* < 0.0001)	0.072
Discharge to facility	0.824 (*p* < 0.001)	0.106
Cardiac complication	0.836 (*p* < 0.0001)	0.081
Pneumonia	0.794 (*p* < 0.001)	0.103
Serious complication	0.772 (*p* < 0.0001)	0.161
Any complication	0.741 (*p* < 0.0001)	0.200
Return to OR	0.738 (*p* < 0.0001)	0.066
SSI	0.729 (*p* < 0.0001)	0.131

*AUC* area under curve,* SSI* surgical site infection

**Fig. 2:**
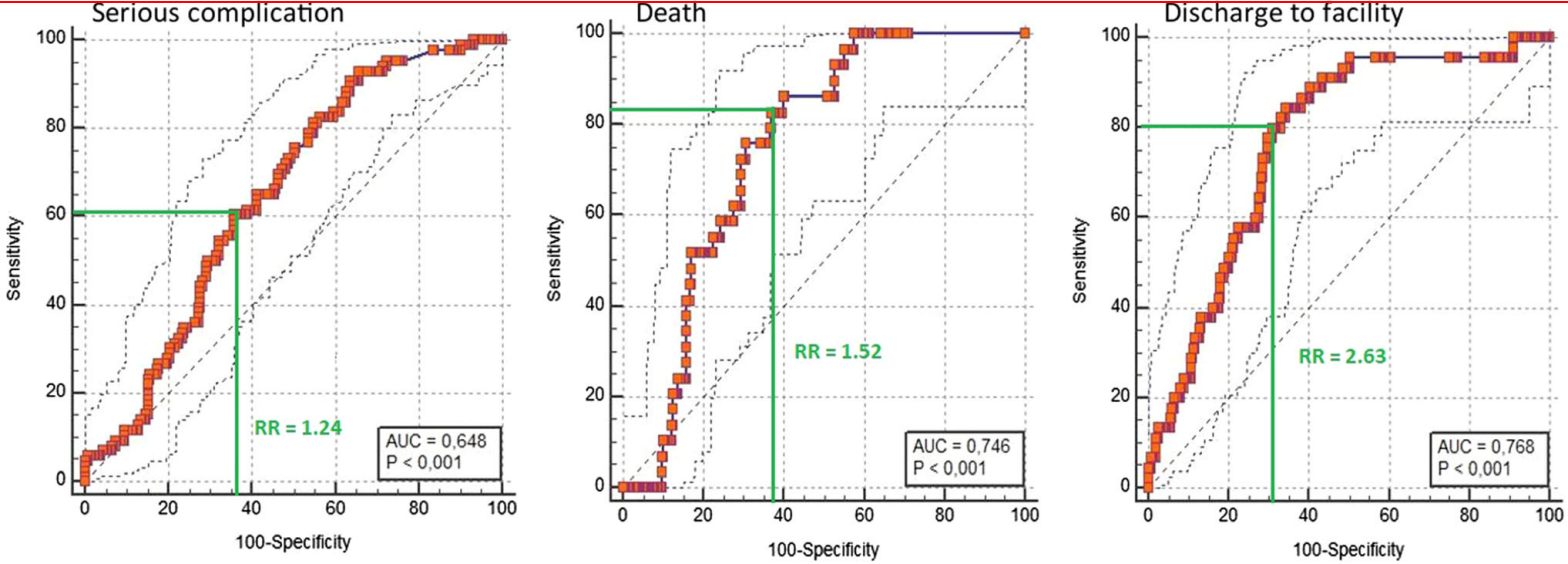
Risk ratio cutoff

The calculated personal RRs for serious complication, death, and discharge to nursing or rehab facility were used to generate three additional ROC curves (Fig. 2). The AUC was 0.648 for serious complication (95%CI 0.593–0.701, *p* < 0.0001), 0.746 for death (95%CI 0.694–0.793, *p* < 0.0001), and 0.768 for discharge to nursing or rehab facility (95%CI 0.718–0.814, *p* < 0.0001).

Three cutoff values in the RR were chosen in order to maximize sensitivity and specificity, keeping both at least > 60%. For serious complication, a RR > 1.24 (RR range 1.18–1.30); the reported range refers to cutoff values whose related sensitivity and specificity are simultaneously greater than 0.6) predicted the event with a sensitivity of 60.47%, a specificity of 64.07%, and a negative predicted value (NPV) of 81%. A RR > 1.52 (RR range 1.01–1.94) and RR > 2.63 (RR range 1.82–3.43) predicted the onset of death (sensitivity = 82.76, specificity = 62.85; NPV = 97%) and discharge to nursing or rehab facility (sensitivity = 80.00, specificity = 69.12; NPV = 95%), respectively (Fig. 2).

## Discussion

The decision-making process is facilitated by an accurate risk estimation, which should be shared between the surgeon and the patient. In the original evaluation of the SRC in 2013, the observed and predicted rates of complications were similar, with satisfactory c-statistics (≥80%) [6]. Although Brier score analysis supported the accuracy of the calculator, the authors did not conduct a detailed evaluation of calibration. A subsequent analysis was run in 2016 on 2.743.679 surgical procedures from 586 ACS-NSQIP participating hospitals and showed a slight tendency of the calculator to overestimate the risk of the lowest and highest risk patients, and underestimate the risk of mid-risk patients. A powerful post-modeling method was therefore used to recalibrate the model, obtaining strict similarity between predicted and observed rates [12].

Since its introduction, the ACS-NSQIP SRC has been tested by several surgeons in different surgical specialties, such as colorectal, pancreatic, reconstruction, gynecologic, orthopedic, urologic, and neurosurgical with variable results [13–19]. However, there is limited literature on the accuracy of the ACS-NSQIP SRC in the acute care surgery. In an emergency surgery setting, the surgeon faces a great variability of situations. Patients with the same diagnosis can present with different clinical scenarios and can quickly deteriorate. Moreover, there is a limited space for an actual preoperative optimization regarding factors that increase morbidity and mortality. For this reason, a great number of variables can influence the accuracy and limit the utility of risk estimations for emergency surgery compared to elective surgery patients [20].

The utility of the surgical risk calculator has been recently studied in a comparison of 56.942 patients who underwent emergency surgeries with 136.311 elective patients, showing a slight underestimation of the risks of emergency surgery compared to the risks of elective surgery (most relevant for gastrointestinal surgeries). The investigators attribute this result to the greater heterogeneity of emergency surgical patients. However, the authors concluded that despite small differences in accuracy of risk prediction, data provide no evidence that would discourage hospitals from applying ACS-NSQIP models for identifying at-risk patients and that the ACS-NSQIP SRC is a potential useful tool for both elective and emergency surgical patients [21]. Burgess et al. [22] studied the predicted versus observed events in a series of 95 patients who underwent emergency laparotomy. The ACS-NSQIP SRC underestimated the risk of serious complications (26% vs. 39%), overall complications (32.4% vs. 45.3%), surgical site infections (9.3% vs. 20%), and length of stay (9.7 days vs. 13.1 days). It accurately predicted the risk of other specific complications and death [22].

Our findings support the effectiveness of the surgical risk calculator even for emergency surgery. We firstly found that the prevalence of observed complications was greater in patients belonging to the group with RR > 1, demonstrating a preliminary capability of the score to identify patients at risk of complication. Discriminative performance determined by AUC was particularly good for death, discharge to nursing or rehab facility, and renal failure, with calculated AUCs above or very close to 0.9. Surgical site infection and return to OR presented the lowest AUCs, but still >0.7 (0.729 and 0.738, respectively). Calibration assessed by the Brier score was informative for all considered outcomes (Brier scores <0.25) except for renal failure (0.689), being particularly significant for death (0.072), return to OR (0.066), and cardiac complication (0.081). The length of stay was accurately predicted, with no statistical differences between the predicted (6.5 days) and the observed (7 days) median values (*p* = 0.695).

One of the aims of this study was the introduction of a RR cutoff to provide a method to weigh the risk in a single patient in a more practical way. For example, we found that a RR > 1.52 predicted the onset of death (sensitivity 82.76%, specificity 62.85%) with a NPV of 97%, thus permitting the almost complete exclusion of death events in all patients with a ratio <1.52. The simple calculation of the RR could help give both the surgeon and the patient an answer in a “yes or no” fashion instead of with a percentage. However, the results of this analysis should be interpreted with caution. Indeed, the ROC curves generated from the RRs have different AUCs when compared to the AUCs generated from the original curves. A possible explanation is that dividing the personal predicted risk by the average risk of a specific surgical procedure, we introduced a further variability. This could be attributable to small differences in average risk provided by the ACS-NSQIP (based on the American population) compared to the average risk of our population. Although not very relevant in our series, this analysis could represent a starting point for further investigation in a larger group of patients.

Our study has limitations. First, our validation analysis used data collected from a single surgical center with a relatively small sample size, which strongly contrasts with the original development of the SRC from over 4.3 million operations of 780 American hospitals. As reported by the Liu et al. [12], “the influence of local quality effects when local quality differs from the NSQIP average.” Second, our study was conducted including different emergency surgical procedures in a single group of analysis. We used 21 CPT codes for a total of 317 patients. Analysis of single subgroups could not be performed due to the paucity of events in each subclass. We should consider that discrimination and calibration results could substantially change between specific types of operations. For example, Golden et al. [9] collected data from 1693 acute care procedures over a 5-year time period. They found that the calculator had a good discriminative power in predicting both serious and any complications rates after acute care surgeries (AUC 0.81 and 0.79, respectively) when considering the whole population as a single group. However, the predictive accuracy varied largely when analyzing different classes of procedures [9]. Even in the original analysis of the ACS, calibration results for specific types of operations resulted sometimes ambiguous, because the small sample sizes contributed to increased variability [12]. Finally, our results emerged from retrospective patient records, in contrast to the ACS-NSQIP database, which derives from prospectively collected data. Inadequate documentation and data collection may have influenced the risk calculator predictions.

## Conclusions

The calculator appears to perform well in predicting adverse postoperative outcomes in our emergency setting, with accurate discrimination and calibration. Discrimination and calibration were particularly good for mortality, discharge to nursing or rehab facility, and cardiac complication. The introduction of a RR cutoff could provide a more practical method to forecast the onset of a specific type of complication in a single patient. The current work should be intended as a preliminary study, which in the future would involve a greater number of surgical centers and patients.
